# hsa-MicroRNA-28-5p Inhibits Diffuse Large B-Cell Lymphoma Cell Proliferation by Downregulating 14-3-3*ζ* Expression

**DOI:** 10.1155/2022/4605329

**Published:** 2022-01-04

**Authors:** Shufang Yan, Yang Chen, Meihong Yang, Qian Zhang, Jiajia Ma, Bo Liu, Liuqing Yang, Xinxia Li

**Affiliations:** ^1^Department of Pathology, The Tumor Hospital, Affiliated to Xinjiang Medical University, Xinjiang 830011, China; ^2^Xinjiang Medical University, Xinjiang 830011, China; ^3^Department of Orthopedics, The First Affiliated Hospital of Tsinghua University, Beijing Huaxin Hospital, Beijing 100016, China

## Abstract

MicroRNAs (miRNAs) participate in the comprehensive biological process of several cancer types. In our former study, we found that hsa-microRNA- (miR-)28-5p was downregulated, but tyrosine 3-monooxygenase/tryptophan 5-monooxygenase activating protein zeta (14-3-3*ζ* or YWHAZ) was upregulated in diffuse large B-cell lymphoma (DLBCL) tissues. We predicted that YWHAZ was a target gene for hsa-miR- 28-5p using bioinformatics analysis. Our goal was to reveal the role of hsa-miR-28-5p in DLBCL. YWHAZ was tested by immunohistochemistry (IHC) in formalin-fixed paraffin-embedded (FFPE) tissues of 137 DLBCL tissues, and the expression of hsa-miR-28-5p and YWHAZ was examined by quantitative real-time polymerase chain reaction (qRT-PCR) in 15 fresh and frozen DLBCL tissues. To study the functional roles of the downregulated hsa-miR-28-5p in DLBCL, a Cell Counting Kit-8 assay was conducted to estimate cell proliferation. Transient transfection of miRNA mimics was performed to overexpress hsa-miR-28-5p, and flow cytometry was performed to examine cell apoptosis and cell cycle progression. A dual-luciferase reporter assay was employed to explore the relationship between hsa-miR-28-5p and YWHAZ. Western blotting and qRT-PCR were used to investigate the function of hsa-miR-28-5p in YWHAZ expression. hsa-miR-28-5p was found to be significantly downregulated in DLBCL tissues and cell lines. Functional studies showed that hsa-miR-28-5p overexpression inhibited cell viability and proliferation, and YWHAZ was predicted to be a target of hsa-miR-28-5p. Dual-luciferase reporter assay, Western blotting, and qRT-PCR verified that hsa-miR-28-5p negatively regulated YWHAZ expression by directly targeting its 3′ untranslated regions in DLBCL cells. hsa-miR-28-5p may suppress the growth of DLBCL cells by inhibiting YWHAZ expression. These findings could provide novel targets for DLBCL diagnosis and therapy.

## 1. Introduction

Diffuse large B-cell lymphoma (DLBCL) is the most common pathological subtype of non-Hodgkin's lymphoma (NHL), accounting for 30%–58% of NHLs [1]. It is also the most common type of lymphoma. In the domestic lymphoma cooperation group, the incidence of DLBCL is as high as 40–50%, which is higher than the international incidence of 30%. DLBCL is a highly heterogeneous and aggressive group of B-cell lymphomas with diverse clinical features, histological morphology, and gene and molecular phenotypes [2]. Although the R-CHOP regimen can significantly improve the prognosis of most patients, there are individual differences among patients in the drug resistance of tumor cells stimulated by chemotherapeutic drugs. One-third of patients relapse within a short period of time after the current treatment regimen and reach the advanced tumor stage [3]. For relapsed/refractory (R/R) DLBCL patients, although salvage autologous hematopoietic stem cell transplantation (ASCT) after chemotherapy can achieve longer-term remission, the significant toxicity of high-dose chemotherapy drugs limits the further treatment of patients with complications and elderly patients. The existing salvage chemotherapy combined with ASCT can cure only approximately 10% of patients [4], and the prognosis of patients diagnosed with R/R DLBCL remains poor. Prolonging the survival time of the remaining 30% of patients and improving their prognosis has become the focus of research at home and abroad. In the last few years, the Ki67 index, International Prognostic Index (IPI) score, serum lactate dehydrogenase (LDH), Ann Arbor stages, and the classification of Han's model have been used clinically and pathologically, but these evaluation systems are unable to analyze patients who do not respond to treatment. Therefore, there is an urgent need for new biomarkers that have better stratification methods to analyze different levels of patients.

The roles of microRNAs (miRNAs) in the development and function of T and B cells have been confirmed [5]. hsa-microRNA- (miR-) 28-5p is an intragenic miRNA that targets a variety of tumor-related genes and participates in cell proliferation, migration, invasion, and epithelial-mesenchymal transformation [6]. The expression of hsa-miR-28-5p in carcinomas is high, low, or lost [6-9], but its expression is downregulated, lost, or upregulated in B-cell lymphoma [7-9]. For example, hsa-miR-28-3p and hsa-miR-28-5p are upregulated in GCB-DLBCL [9]. Recent research showed that high expression of hsa-miR-28-5p hampers tumor growth in DLBCL [8]. Previous studies have characterized the genes regulated by hsa-miR-28 in other human B-cell lymphoma cells by transcriptomic and proteomic analysis [8]. However, the exact mechanism of hsa-miR-28-5p in DLBCL is not clearly known.

Our research group showed that hsa-miR-28-5p was downregulated in DLBCL tissues (GSE173080) using an Agilent Human miRNA Microarray [10]. The series record GSE173080 provides access to all of the current data (https://www.ncbi.nlm.nih.gov/geo/query/acc.cgi?acc=GSE173080). Furthermore, we found that the protein tyrosine 3-monooxygenase/tryptophan 5-monooxygenase activating protein zeta (also named 14-3-3*ζ* or YWHAZ) was differentially expressed based on isobaric tags for relative and absolute quantitation (iTRAQ). Parallel reaction monitoring (PRM) verified that YWHAZ was upregulated in DLBCL tissues. The high expression of YWHAZ was related to a poor prognosis of DLBCL [11]. According to the TargetScan database (http://www.targetscan.org) and starBase (https://starbase.sysu.edu.cn), YWHAZ is a direct target of hsa-miR-28-5p. We predicted that the hsa-miR-28-5p/YWHAZ axis might be involved in the pathogenesis of DLBCL. However, no studies have evaluated how hsa-miR-28-5p relates to the occurrence and development of DLBCL.

## 2. Materials and Methods

### 2.1. Tissue Samples

The study was approved by the Medical Ethics Committee of the Tumor Hospital Affiliated to Xinjiang Medical University, and 15 patients and/or their families signed informed consent before the histological biopsy was carried out by the patients' chief physician. Fresh and frozen DLBCL samples were stored at −80°C, other samples were from formalin-fixed paraffin-embedded (FFPE) tissues, and treatments were not given before this research. The features of 15 patients with fresh and frozen samples are described in Table 1.

Features of explanation:^∗^*p* < 0.05 represents significant differences. GCB subtype, germinal center B-cell subtype; ABC subtype, activated B-cell-like subtype; LDH, lactate dehydrogenase; YWHAZ, 3-monooxygenase/tryptophan 5-monooxygenase activating protein zeta (also named 14-3-3*ζ*); DLBCL, diffuse large B-cell lymphoma.

### 2.2. Tissue Microarray and Immunohistochemistry (IHC)

Three tissue microarray (TMA) blocks were constructed using a tissue arrayer. Each individual case was represented by two tumor cores with a diameter of 0.6 mm that had been taken from the original paraffin blocks. Serial sections of 3 µm were prepared from the tissue array blocks and used in the immunohistochemical analysis, including YWHAZ (ab51129, Abcam, 1 : 100, cytoplasm). Pretreatment in 1 mm Tris/EDTA buffer (pH 9.0, no. ZLI-9069, ZSBIO, China) was conducted for 25 minutes at 98°C. Using 3% hydrogen peroxide to block nonspecific peroxidase reactions, tissues were placed in a humidified incubator at 37 C for 20 minutes. After washing three times with phosphate-buffered saline (PBS) for 5 minutes each time, the tissues were dried, and a small amount of YWHAZ monoclonal antibody (1 : 100, Abcam, ab51129) was added at 4°C overnight. The tissues were incubated with rabbit anti-mouse IgG antibody (PV-6000, ZSBIO, China) for 20 minutes at room temperature as a secondary antibody application. The staining intensity of tumor tissues was scored as 0 (negative), 1 (weak), 2 (moderate), or 3 (strong) for antigens present in the cytoplasm using a light microscope (magnification, ×400), determined separately by two independent pathologists. DLBCL was classified according to Han's algorithm: the germinal center B-cell (GCB) subtype had a CD10+ or CD10−, Bcl-6+, and MUM1− phenotype, whereas the Bcl-6- or Bcl-6+, CD10−, and MUM1+  phenotype represented the non-GCB subtype (including the activated B-cell-like (ABC) subtype and unclassified subtype in this study). IHC was performed following the manufacturer's protocols.

### 2.3. Cell Lines

Human DLBCL cell lines were Su-DHL-2 (non-GCB), SU-DHL-6 (GCB), IM-9 (human normal peripheral blood B-lymphocytes line), OCI-LY1 (GCB), and OCI-LY3 (non-GCB). OCI-LY1 was provided by the Institute of Jennio Biotech (CBP0265, Guangzhou, China). OCI-LY3 cells were obtained from the Institute of YaJi Biotechnology (YS1021 C, Shanghai, China). Su-DHL-2 (KG541, Nanjing, China) and SU-DHL-6 from KeyGen Biotechnology (KG542, Nanjing, China) cells were acquired. IM-9 (YS329 C) was obtained from YaJi Biotechnology. Cells were cultured in Roswell Park Memorial Institute-1640 (RPMI-1640) medium (C118775500CP, Gibco, USA) supplemented with 10% fetal bovine serum (FBS) (FND500, Excell Bio, USA) and 1% penicillin-streptomycin (PS) (10000U, GIBCO, USA). All cells were placed in a humidified incubator containing 5% CO2 and a 95% air atmosphere at 37°C.

The human embryonic kidney cell line HEK-293T was obtained from Procell Life Science and Technology (CL-0005, Wuhan, China). The lentivirus packaging cell line HEK-293 TN was sustained in Dulbecco's modified Eagle medium (DMEM) (C11995500BT, Gibco, USA) supplemented with 10% FBS and 1% PS (10000U, 15070–063, Gibco, USA). The HEK-293T cell line was only applied to the dual-luciferase reporter assay and packaging lentivirus. All cells were placed in a humidified incubator at 37°C with 5% CO2. Cells were passaged until up to 90% of the cells were fused to the wall.

### 2.4. Cell Transfection

The YWHAZ overexpression plasmid and siRNAs against YWHAZ were purchased from General Biol (Anhui, China), with scramble plasmid and siRNA employed as negative controls. hsa-miR-28-5p mimics and hsa-miR-28-5p inhibitors were synthesized by Gene Pharma (Suzhou, China). All of the above reagents were transfected into cells via Lipofectamine 3000® Transfection Reagent (L3000-008, Invitrogen, USA) according to the manufacturer's recommendations. The transfection conditions were as follows: hsa-miR-28-5p mimics or inhibitor negative control labeled with 0.1 *μ*m red fluorescence was transfected into cells 48 hours after transfection. Under a fluorescence microscope, the transfection efficiency was determined by naked eye observation of the red fluorescence expression, and the transfection efficiency was more than 70%.

### 2.5. Luciferase Reporter Gene Assay

Using Lipofectamine 3000® Transfection Reagent, 293T cells at logarithmic phase were cotransfected with mimic-28-5p (Gene Pharma, Suzhou, China), con-28-5p (Gene Pharma, Suzhou, China), and a luciferase reporter vector including the wild-type (WT) (General bio, Anhui, China) 3' untranslated regions (3' UTR) or mutant-type (MT) (General bio, Anhui, China) 3' UTR. After transfection for 48 hours, the luciferase activity was measured by a dual-glo-luciferase assay system (E2920, Promega, Madison, USA) following the manufacturer's protocol.

### 2.6. Silencing of YWHAZ

To choose the most effective siRNA for silencing YWHAZ, we selected three options: YWHAZ-Homo (Y206) (Gene Pharma, Suzhou, China), YWHAZ-Homo (Y319) (Gene Pharma, Suzhou, China), and YWHAZ-Homo (Y778) (Gene Pharma, Suzhou, China). After verification by qRT-PCR, siRNA-YWHAZ (Y319) was used for the experiment.

### 2.7. Quantitative Real-Time Polymerase Chain Reaction (qRT-PCR)

Total RNA was extracted from DLBCL cells using TRIzol reagent (15596026, Ambion, USA). Reverse transcription into cDNA was performed with miRNA First-Strand cDNA Synthesis (Tailing Reaction) (B532451, Sangon Biotech, Shanghai, China) according to the manufacturer's instructions. Glyceraldehyde-3-phosphate dehydrogenase (GAPDH) was used as an internal standard. The sequences of PCR primers are shown as follows: YWHAZ forward primer: 5'-TGTAGGAGCCCGTAGGTCATC-3'; YWHAZ reverse primer: 5'-GTGAAGCATTGGGGATCAAGA-3'; GAPDH forward primer: 5'-TGTTGCCATCAATGACCCCTT-3'; GAPDH reverse primer: 5'-CTCCACGACGTACTCAGCG-3'; hsa-miR-28-5p forward primer: 5'-CGCAGCACTAGATTGTGA-3'. The concentration and 260/280 of total RNA extracted from experimental cell samples were between 569.55 ng/ml and 831.70 ng/ml and between 1.8 and 2.0, respectively. RNA quality detected by agarose electrophoresis strips was measured. Expression levels of hsa-miR-28-5p and YWHAZ mRNA were detected in SU-DHL-2, SU-DHL-6, IM-9, OCI-LY1, and OCI-LY3 cell lines.

### 2.8. Western Blotting Assay

Protein was extracted from cells and transferred to polyvinylidene fluoride (PVDF) membranes after sodium dodecyl sulfate-polyacrylamide gel electrophoresis (SDS-PAGE). Then, membranes were blocked with 5% nonfat milk for 1 hour and incubated overnight at 4° C with the following primary antibodies: YWHAZ (1 : 500, Abcam, ab51129) and *β*-actin (38 KD, Sino Biological Inc, 100166-MM1). After washing the membranes three times with Tris Buffered Solution Tween (TBST), a secondary antibody was incubated with the membranes for 1 hour at room temperature. The dilution ratio was determined according to the instructions.

### 2.9. Cell Counting Kit-8 (CCK-8)

Cell proliferation analysis was established using the CCK-8 reagent (FC101-03, Beijing, China). OCI-LY1 cells were seeded into 96-well plates at 5*∗*10^3^ cells/well. At the indicated time points, 10 *μ*l CCK-8 was supplemented, and the cells were incubated for 1 hour at 37°C. The optical density was identified at 450 nm with a microplate spectrophotometer.

### 2.10. Flow Cytometry Assays

Cell cycle analysis was performed using flow cytometry (LSRFortessa, BD, USA). The cells were resuspended in 500 µl of PBS (ZLI-9062, Beijing, China) and 3.5 ml of anhydrous ethanol for fixation overnight. Cells (1*∗*10^6^) were separated by centrifugation at 2,000 rpm for 5 minutes and accumulated and washed twice with cold PBS. Then, 500 µl of PI/RNase (550825, BD, USA) was added to resuspend the cells. After passing through a 200 Mesh Nylon screen, single-cell suspensions were prepared, and the sample was incubated at 4° C in the dark for 30 minutes. Flow cytometry was used to detect red fluorescence at 488 nm and light scattering. DNA content analysis and light scattering analysis were carried out with analysis software. For apoptosis analysis, cells were collected 48 hours after transfection. Cells were double-stained using a FITC Annexin V PE/7AAD Kit (559763, BD, USA). Then, cells were detected by flow cytometry after 30 minutes.

### 2.11. Statistical Analysis

SPSS 23.0 statistical software and GraphPad Prism 8.0 software were applied for data analysis. All data are presented as the means plus or minus standard deviation. Student's *t*-test, one-way ANOVA, and Fisher's exact test were used to perform statistical analysis. *p* < 0.05 was considered statistically significant.

## 3. Results

### 3.1. hsa-miR-28-5p Is Downregulated and YWHAZ Is Upregulated in Human DLBCL Tissues

**Figure 1:**
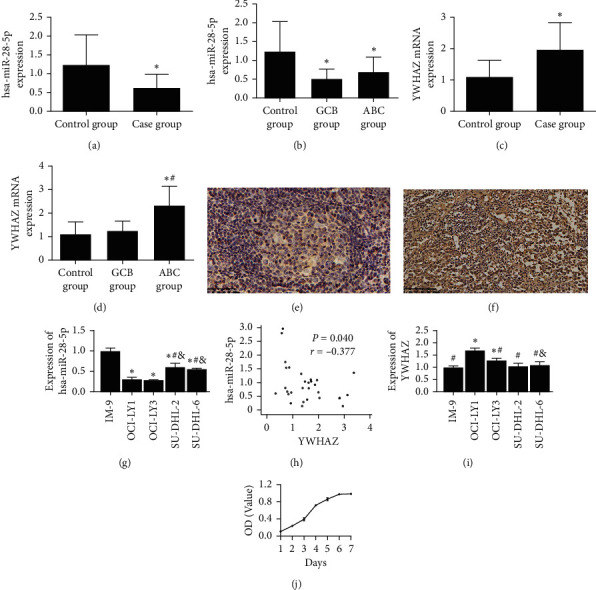
Downregulation of hsa-miR-28-5p and upregulation of YWHAZ in fresh tissues of DLBCL patients were tested by qRT-PCR. (a) Expression levels of hsa-miR-28-5p in the case group were lower than those in the control group (reactive hyperplasia of lymph nodes group). (b) Expression levels of hsa-miR-28-5p in the GCB group were lower than those in the ABC group. (c) Expression levels of YWHAZ in human DLBCL tissues were higher than those in reactive hyperplasia of lymph nodes. (d) Expression levels of YWHAZ in the ABC group were higher than those in the GCB group. (e) (EnVision method, original magnification x400) In reactive hyperplasia of lymph nodes tissue, YWHAZ expressed negatively. (f) (EnVision method, original magnification x400) YWHAZ expressed on oncocytes: the YWHAZ expression was positive in the cytoplasm and cell membrane of the tumor cells. (a–d) *∗p* < 0.05 versus the control group; #*p* < 0.05 versus the GCB group. (g) Expression levels of hsa-miR-28-5p were lower in DLBCL cell lines. (h) YWHAZ was negatively correlated with hsa-miR-28-5p, and the correlation coefficient was −0.377 (*p*=0.040). (i) Expression levels of YWHAZ were higher in DLBCL cell lines. (j) The OCI-LY1 cell line grew well, and it was chosen for our experiment. (g, i) *∗p* < 0.05 versus the IM-9 group; #*p* < 0.05 versus the OCI-LY1 group; & *p* < 0.05 versus the OCI-LY3 group (one-way ANOVA).

The expression levels of hsa-miR-28-5p and YWHAZ in DLBCL cell lines were detected by qRT-PCR, and the DLBCL cell lines for subsequent cell function tests were identified. To study the roles of hsa-miR-28-5p in the development of DLBCL, the expression patterns of hsa-miR-28-5p were analyzed by qRT-PCR. We chose reactive hyperplasia of lymph nodes for our experiment. As shown in Figures 1(a)–1(d), hsa-miR-28-5p was downregulated, and YWHAZ was upregulated in DLBCL tissues.  Figure 1(d) shows that the ABC group had a higher expression of YWHAZ than the other groups in fresh and frozen DLBCL tissues. YWHAZ immunoreactivity was not observed in reactive hyperplasia of lymph nodes tissue (Figure 1(e)). Heavy and diffuse YWHAZ immunoreactivity was observed in the cytoplasm of DLBCL cells (Figure 1(f)). The relative expression of hsa-miR-28-5p was the highest in the IM-9 cell line and clearly decreased in DLBCL cell lines; the difference was statistically significant (Figure 1(g)). YWHAZ was significantly negatively correlated with hsa-miR-28-5p (Figure 1(h)). The relative expression of YWHAZ was the lowest in IM-9 cells and clearly increased in DLBCL cell lines; the difference between the OCI-LY1 group and the OCI-LY3 group was statistically significant (Figure 1(i)). Based on the above results, we identified OCI-LY1 cells for subsequent cell function studies.  Figure 1(j) shows the growth of OCI-LY1 cells.

### 3.2. YWHAZ Is a Direct Target Gene of hsa-miR-28-5p

**Figure 2:**
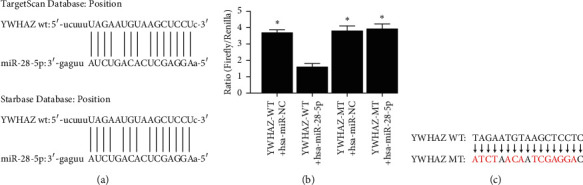
YWHAZ is a direct target gene of hsa-miR-28-5p. (a) Potential interacting sites of hsa-miR-28-5p and YWHAZ were predicted by bioinformatics tools. (b) Results of double luciferase reporter gene verification of the interaction between hsa-miR-28-5p and the YWHAZ gene. (c) The mutation points for YWHAZ. Red represents the mutation points for YWHAZ. *∗p* < 0.05 versus the YWHAZ-WT + hsa-miR-28-5p group (one-way ANOVA).

To ascertain the detailed regulatory mechanism of hsa-miR-28-5p in DLBCL, we searched the TargetScan and StarBase databases and observed that YWHAZ was predicted to be a downstream target of hsa-miR-28-5p (Figure 2(a)). The luciferase activity in the YWHAZ-WT + hsa-miR-28-5p group was significantly lower than that in the other three groups (Figure 2(b)). The 3'-UTR of the YWHAZ gene and hsa-miR-28-5p complemented each other, and their binding caused the fluorescence expression of the plasmid to decrease; however, the fluorescent expression of the plasmid did not change after cotransfection with the reporter plasmid of hsa-miR-28-5p and YWHAZ's 3'-UTR mutant sequence. The results showed that hsa-miR-28-5p could interact with YWHAZ by binding to the 3'-UTR of YWHAZ (Figure 2(b)), indicating that YWHAZ is a target gene regulated directly by hsa-miR-28-5p. The mutation points for YWHAZ are shown in Figure 2(c).

### 3.3. Screening the Best Appropriate siRNA and Verifying the Effect of Silencing YWHAZ

**Figure 3:**
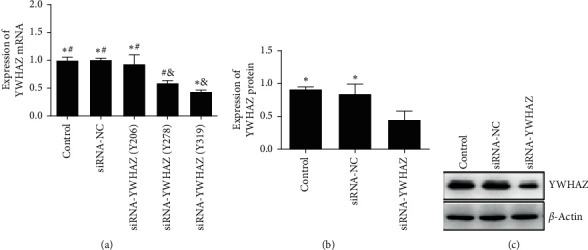
Screening the best appropriate siRNA and verifying the effect of silencing YWHAZ. (a) Expression level of YWHAZ was the lowest in siRNA-YWHAZ (Y319). (b) Western blotting verified that siRNA-YWHAZ possessed a good silencing effect. (c) The siRNA-YWHAZ group had the lowest expression level of YWHAZ; *∗p* < 0.05 versus the siRNA-YWHAZ (Y278) group or the siRNA-YWHAZ group; #*p* < 0.05 versus the siRNA-YWHAZ (Y319) group; &*p* < 0.05 versus the control group (one-way ANOVA).

qRT-PCR was used to choose the most proper siRNA-YWHAZ (Figures 3(a)–3(c)). The concentration, 260/280, and quality of total RNA extracted from experimental cell samples were measured. We selected a 0.1 *μ*m siRNA transfection concentration, and 48 hours was used as the best transfection conditions for follow-up experiments.  Figure 3(a) shows that the siRNA-YWHAZ (Y319) group could effectively inhibit the gene expression of YWHAZ. Western blotting analysis was used to confirm the effect of silencing YWHAZ (Figures 3(b) and 3(c)). The concentration and quality of the protein extracted from the experimental cell samples were both satisfactory. The protein level of YWHAZ in the siRNA-YWHAZ group was lower than that in the other two groups. SiRNA-YWHAZ suppressed the protein level of YWHAZ (Figures 3(b) and 3(c)).

### 3.4. Mechanism by Which hsa-miR-28-5p Targets YWHAZ and Affects DLBCL Cell Proliferation, Apoptosis, and Cell Cycle Progression

**Figure 4:**
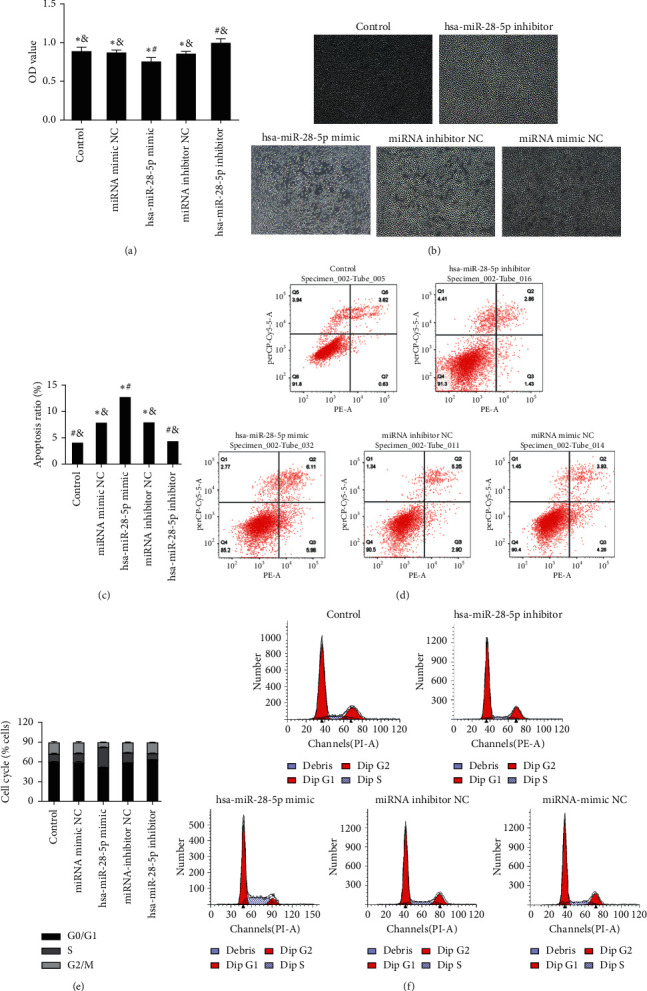
The mechanism by which hsa-miR-28-5p targets YWHAZ affecting the cell function of DLBCL and the effects of hsa-miR-28-5p on the proliferation, apoptosis, and cell cycle of DLBCL cells. (a, b) A CCK-8 assay was used to test cell proliferation, x100. (c, d) Cells were double-stained using a FITC Annexin V PE/7AAD Kit to determine the cell apoptosis rate. (e, f) The cell cycle was detected by flow cytometry PI staining. *∗p* < 0.05 versus the hsa-miR-28-5p inhibitor group; #*p* < 0.05 versus the hsa-miR-28-5p inhibitor NC group; &*p* < 0.05 versus the hsa-miR-28-5p mimic group (one-way ANOVA).

**Figure 5:**
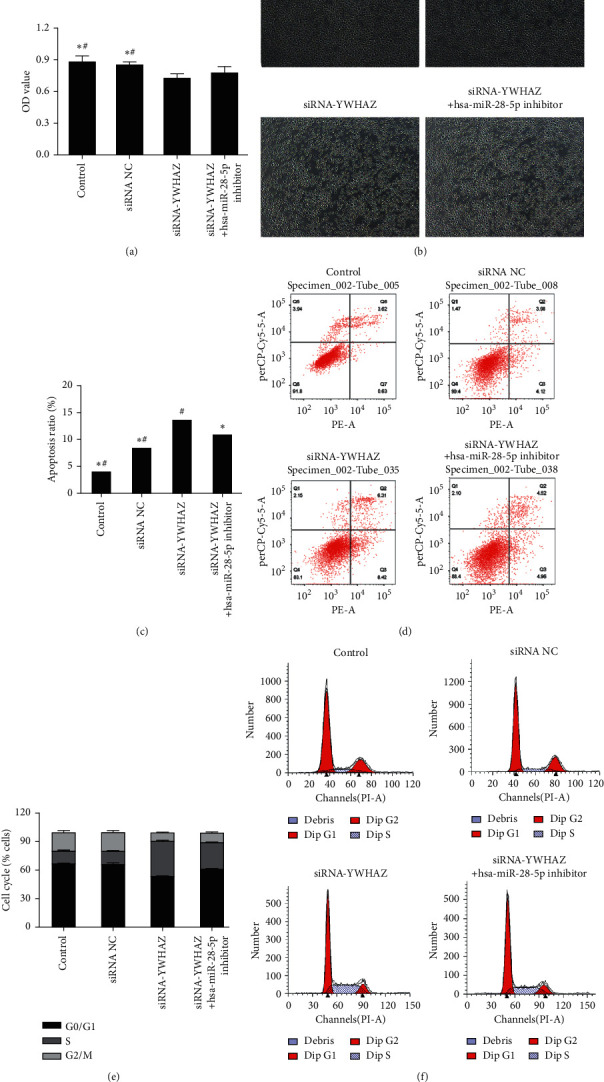
The mechanism by which hsa-miR-28-5p targets YWHAZ affecting the cell function of DLBCL and the effects of YWHAZ on the proliferation, apoptosis, and cell cycle of DLBCL cells. (a, b) A CCK-8 assay was used to test cell proliferation, x100. (c, d) Cells were double-stained using a FITC Annexin V PE/7AAD Kit to determine the cell apoptosis rate. (e, f) The cell cycle was detected by flow cytometry PI staining. *∗p* < 0.05 versus the siRNA-YWHAZ group; #*p* < 0.05 versus the siRNA-YWHAZ group + hsa-miR-28-5p inhibitor group (one-way ANOVA).

Compared with the miRNA mimic NC group and control group, overexpression of hsa-miR-28-5p (Figures 4(a) and 4(b)) in OCI-LY1 cells led to lower proliferation as determined by CCK-8. Increased apoptosis (Figures 4(c) and 4(d)) and a significantly increased number of cells in the S phase were observed by flow cytometry (Figures 4(e) and 4(f)). Compared with the siRNA-NC group and control group, silencing the YWHAZ gene (Figures 5(a) and 5(b))) in OCI-LY1 cells led to lower proliferation as determined by CCK-8, increased apoptosis (Figures 5(c) and 5(d)), and a significantly increased number of cells in S phase (Figures 5(e) and 5(f)).

**Figure 6:**
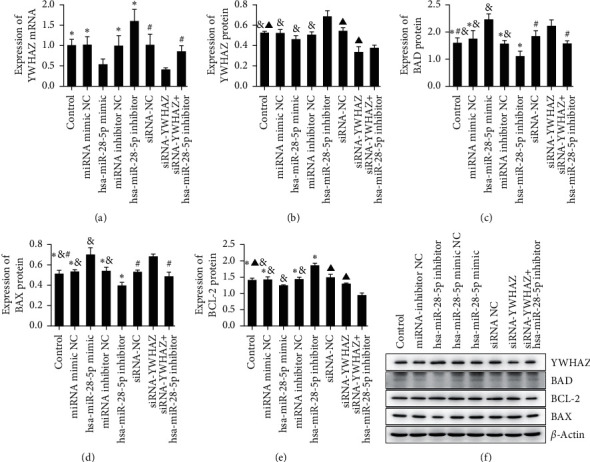
Apoptosis-related protein and gene levels were assessed by Western blotting and qRT-PCR, respectively. (a) The gene expression levels of YWHAZ were detected by qRT-PCR. (b–e) The protein expression levels of YWHAZ, BAX, BAD, and BCL-2 were determined by Western blotting. (f) hsa-miR-28-5p and YWHAZ affected the protein expression levels of BAX, BAD, and BCL-2. ^*∗*^*p* < 0.05 versus the hsa-miR-28-5p mimic; #*p* < 0.05 versus siRNA-YWHAZ; &*p* < 0.05 versus the hsa-miR-28-5p inhibitor; ▲*p* < 0.05 versus the siRNA-YWHAZ + hsa-miR-28-5p inhibitor group (one-way ANOVA).

As shown in our experiments, apoptosis-related protein and gene expression levels were determined by Western blotting and qRT-PCR, respectively. The results indicated that the gene and protein levels of YWHAZ were higher in the siRNA-YWHAZ + hsa-miR-28-5p inhibitor group than in the siRNA-YWHAZ group (Figures 6(a) and 6(b)). Compared with the control group, miRNA mimic NC group, miRNA inhibitor NC group, and hsa-miR-28-5p inhibitor group, overexpression of hsa-miR-28-5p significantly increased the protein levels of the cleaved proapoptotic proteins BAD and BAX (Figures 6(c) and 6(d)) and decreased levels of the antiapoptotic protein BCL-2 in OCI-LY1 cells (Figure 6(e)). A subsequent comparison with the control group, siRNA-NC group, siRNA-YWHAZ + hsa-miR-28-5p inhibitor group, and siRNA-YWHAZ group obtained the same result (Figures 6(c)–6(e))). In conclusion, siRNA-YWHAZ inhibits the transcription and translation of YWHAZ, suppresses proliferation, promotes apoptosis, and inhibits proliferation by regulating the cell cycle of DLBCL cells.

### 3.5. Effect of siRNA-YWHAZ + hsa-miR-28-5p Inhibitor on DLBCL Cells

The CCK-8 results showed that the survival rate of DLBCL cells in the siRNA-YWHAZ group was 82.973%, which was significantly lower than that in the siRNA-NC group (96.708%). However, the cell survival rate of the siRNA-YWHAZ + hsa-miR-28-5p inhibitor group was 88.302%, which was significantly higher than that in the siRNA-YWHAZ group, although there was no clear difference. The results showed that silencing YWHAZ could inhibit the proliferation of DLBCL cells, but the hsa-miR-28-5p inhibitor could reverse the effect induced by YWHAZ silencing on the proliferation inhibition of DLBCL cells (Figures 5(a) and 5(b)).

The total apoptosis rate in the siRNA-YWHAZ group was 13.820 ± 0.788, which was significantly higher than that in the siRNA-NC group (8.690 ± 0.847%), and the difference was statistically significant. The total apoptosis rate of the siRNA-YWHAZ + hsa-miR-28-5P inhibitor group was 11.150 ± 1.447%, which was significantly lower than that of the siRNA-YWHAZ group. These results suggested that silencing YWHAZ can promote apoptosis of DLBCL cells, but an hsa-miR-28-5p inhibitor can reverse this effect in DLBCL cells (Figures 5(c) and 5(d)).

The percentage of S phase cells in the siRNA-YWHAZ + hsa-miR-28-5p inhibitor group (27.627 ± 0.599%) was lower than that in the siRNA-YWHAZ group (36.893 ± 0.602%). These results suggested that the hsa-miR-28-5p inhibitor can reverse the regulatory effect of siRNA-YWHAZ on the DLBCL cell cycle, and YWHAZ can inhibit the proliferation of DLBCL cells by regulating the cell cycle. Inhibiting hsa-miR-28-5p on this basis could reverse this regulatory effect and promote DLBCL cell proliferation (Figures 5(e) and 5(f)).

The results indicated that the gene and protein levels of YWHAZ are higher in the siRNA-YWHAZ + hsa-miR-28-5p inhibitor group than in the siRNA-YWHAZ group (Figures 6(a) and 6(b)). We concluded that the hsa-miR-28-5p inhibitor can reverse the inhibition of YWHAZ transcription and translation induced by siRNA-YWHAZ.

Therefore, the hsa-miR-28-5p inhibitor could partially reverse the effect of siRNA-YWHAZ. These results suggested that siRNA-YWHAZ can inhibit the activation of DLBCL. hsa-miR-28-5p affects DLBCL cell proliferation, apoptosis, and cell cycle progression by targeting YWHAZ.

## 4. Discussion

miRNAs, small and endogenous noncoding RNA molecules with a length of approximately 22 nucleotides, are partially located in tumor-related sites or fragile regions. miRNAs are highly sequential, conserved, and tissue-specific. miRNAs influence comprehensive biological processes by causing targeted degradation or translation inhibition through binding the 3' UTR of a target mRNA [12]. miRNAs are involved in the basic pathways of B-cell development, such as B-cell receptor signaling (BCR) and B-cell migration/adhesion, and affect B-cell maturation and the production of marginal zone, follicular, plasma, and memory B cells [13]. miRNAs may function as oncogenes, tumor suppressors, or both, depending on the tumor environment [14]. hsa-miR-28 expression can downregulate downstream effectors of BCR signaling, downstream effectors have great effects on B lymphocyte proliferation and survival, and their expression is upregulated in germinal center-derived malignant tumor cells [8].

The expression of hsa-miR-28-5p inhibits the proliferation of B-cell lymphoma and renal cell carcinoma cells by regulating the expression of BAG1 [7] and RAP1B, respectively [15]. hsa-miR-28-5p also restrains the migration and invasion of gastric cancer cells by inhibiting AKT [16]. Although the expression of hsa-miR-28-5p is downregulated in B-cell lymphoma, renal cell carcinoma, hepatocellular carcinoma, and colorectal cancer [7, 17–19], the expression level of hsa-miR-28-5p is increased in ovarian, esophageal, and cervical cancer [6, 20, 21]. Most articles regarding the role of hsa-miR-28-5p in tumors indicate that miRNA has universal inhibitory activity in vitro, consistent with a previous article [7], and the finding that reexpression of hsa-miR-28 can damage tumor growth in several lymphoma models [8] verified that the reexpression of hsa-miR-28-5p in a DLBCL xenotransplantation model hampers tumor growth [8]. These findings opened the way for hsa-miR-28-5p-based replacement therapy as a new treatment strategy for DLBCL. The above conclusion is inconsistent with another prior report [9]. According to the other theory, hsa-miR-28 expression could decrease the proliferation and survival of both primary and tumor B lymphocytes, likely by inhibiting BCR signaling [8]. Considering that YWHAZ is located downstream of the BCR signaling pathway and that YWHAZ is the target gene of hsa-miR-28-5p as predicted from the database, we can study the mechanism of hsa-miR-28 in DLBCL by analyzing its regulation of YWHAZ.

Accumulated research has revealed the mechanism of hsa-miR-28-5p in the initiation and development of cancers, including ovarian cancer, glioma, and hepatocellular carcinoma [6, 15, 18], but there are no reports on DLBCL. Functional experiments have shown that the hsa-miR-28-5p inhibitor promotes DLBCL cell proliferation, restrains cell apoptosis, and leads to an abnormal cell cycle. In the present work, we found that YWHAZ acted as the target of hsa-miR-28-5p. In addition, hsa-miR-28-5p was expressed at low levels in DLBCL cell lines. We found that hsa-miR-28-5p could partially reverse the YWHAZ-induced oncogenic effects on DLBCL cells, which was consistent with the previously discovered tumor suppressive roles of hsa-miR-28-5p in other tumors [7, 17–19].

14-3-3 is a family of acidic and dimeric proteins that are profoundly conserved and broadly expressed in eukaryotic cells. Seven 14-3-3 isoforms (*β*, *γ*, *ζ*, *σ*, є, т, and *η*) have been discovered in human cells. YWHAZ, which is part of the 14-3-3 protein family, can perform primarily by binding to clarified phosphoserine/phosphothreonine-containing motifs in protein targets to mediate signaling pathways and regulate many biological processes, including protein transport, metabolism, cell proliferation, migration, apoptosis, and cell cycle regulation. YWHAZ was shown to be upregulated frequently and to function as an oncogene by regulating multiple signaling pathways in tumors [22]. Overexpression of YWHAZ is regulated by miRNAs or long noncoding RNAs. Overexpression of YWHAZ activates downstream molecules, including protein kinases, apoptotic proteins, and metastasis-related molecules, and ultimately promotes the malignant potential of cancer cells [22]. The increased expression of YWHAZ promotes the proliferation, migration, and resistance to apoptosis of prostate cancer cells, while the downregulation of YWHAZ significantly affects the invasiveness of tumor cells [23, 24]. Overexpression of YWHAZ can also be found in adenocarcinoma of the esophagogastric junction, lung cancer, and intrahepatic cholangiocarcinoma [25–27]; thus, overexpression of YWHAZ is related to invasiveness and drug resistance in multiple types of tumors. Our data showed that YWHAZ is a direct target gene of hsa-miR-28-5p and is overexpressed in DLBCL cell lines, which was in line with its oncogenic role in other tumors [23, 25, 27]. Furthermore, the expression of siRNA-YWHAZ inhibited proliferation, promoted apoptosis, and caused the cell cycle to remain in the S phase in DLBCL cells, all of which were reversed by the hsa-miR-28-5p inhibitor. Our investigation demonstrates the underlying mechanism of hsa-miR-28-5p in the initiation and development of DLBCL. BV02 is a nonpeptide inhibitor of the 14-3-3/c-Abl protein-protein interaction, its bioactive form is phthalimide derivative 9, and inhibitors of 14-3-3 protein-protein interaction derived from BV02 are chemically stable [28]. Inosine monophosphate (IMP), pyridoxal phosphate (PLP), and the derivatives show inhibitory action of the 14-3-3/c-Abl PPI poorly [29]. So far, 14-3-3 inhibitors have mono- and bivalent forms [30].

## 5. Conclusions

In summary, we found an oncogenic role for YWHAZ and an antioncogenic role for hsa-miR-28-5p in the proliferation and apoptosis of DLBCL cells. Moreover, our research suggests that hsa-miR-28-5p may suppress the growth of DLBCL cells by inhibiting YWHAZ expression. These findings could provide novel targets for DLBCL diagnosis and therapy.

## Figures and Tables

**Table 1:** Features of 15 patients diagnosed with DLBCL with fresh and frozen samples.

Item	GCB group	ABC group	*p* value
N	5	10	–

*Age (years)*			
<50	2 (2/5, 40)	5 (5/10, 50)	1.000
>50	3 (3/5, 60)	5 (5/10, 50)	

*Gender, n (%)*			
Male	3 (3/5, 60)	8 (8/10, 80)	0.560
Female	2 (2/5, 40)	2 (2/10, 20)	

*Primary localization, n (%)*			
Intranodal	4 (5/5, 80)	6 (6/10, 60)	0.600
Extranodal	1 (1/5, 20)	4 (4/10, 40)	

*Laboratory parameter, mean ± SD (10^9^/l)*			
Leukocytes	10.45 ± 4.01	5.91 ± 3.33	0.036∗
Hemoglobin	121.80 ± 22.20	103.90 ± 26.47	0.218
Thrombocyte	264.80 ± 110.25	119.60 ± 89.30	0.016∗
*LDH increased, n (%)*	2 (2/5, 40)	4 (4/10, 40)	1.000
*B-symptoms, n (%)*	1 (1/5, 20)	4 (4/10, 40)	0.007∗

*Ann Arbor stage, n (%)*		
I∼II (early stage)	3 (3/5, 60)	2 (2/10,20)	0.251
III∼IV (late stage)	2 (2/5, 40)	8 (8/10, 80)	
*Bone marrow involvement, n (%)*	0 (0)	2 (2/10, 20)	0.007∗
*hsa-miRNA-28-5p, mean ± SD*	0.502 ± 0.274	0.699 ± 0.382	0.566
*YWHAZ, mean ± SD*	1.252 ± 0.407	2.331 ± 0.811	0.004∗
*Overall survival, n (%)*	3 (3/5, 60)	8 (8/10, 80)	0.353

## Data Availability

All data generated or analyzed during this study are included in this published article.

## Abbreviations

miR: MicroRNA
DLBCL: Diffuse large B-cell lymphoma
IHC: Immunohistochemistry
FFPE: Formalin-fixed paraffin-embedded
qRT-PCR: Quantitative real-time polymerase chain reaction
NHL: Non-Hodgkin's lymphoma
R/R: Relapsed/refractory
ASCT: Autologous hematopoietic stem cell transplantation
LDH: Lactate dehydrogenase
iTRAQ: Isobaric tags for relative and absolute quantitation
PRM: Parallel reaction monitoring
GCB subtype: Germinal center B-cell subtype
ABC subtype: Activated B-cell-like subtype
3' UTR: 3' untranslated regions
LDH: Lactate dehydrogenase
FBS: Fetal bovine serum
PS: Penicillin-streptomycin
HEK-293T: Human embryonic kidney cell line
DMEM: Dulbecco's modified Eagle medium
GAPDH: Glyceraldehyde 3-phosphate dehydrogenase
PVDF: Polyvinylidene difluoride
CCK-8: Cell Counting Kit-8
NC: Negative control
TBST: Tris Buffered Solution Tween
PBS: Phosphate-buffered saline.
